# Transparency-Aware Segmentation of Glass Objects to Train RGB-Based Pose Estimators

**DOI:** 10.3390/s24020432

**Published:** 2024-01-10

**Authors:** Maira Weidenbach, Tim Laue, Udo Frese

**Affiliations:** Faculty of Mathematics and Computer Science, University of Bremen, 28359 Bremen, Germany; maiweide@uni-bremen.de (M.W.); tlaue@uni-bremen.de (T.L.)

**Keywords:** neural networks, training data, transparent objects, bounding box, segmentation, pose estimation

## Abstract

Robotic manipulation requires object pose knowledge for the objects of interest. In order to perform typical household chores, a robot needs to be able to estimate 6D poses for objects such as water glasses or salad bowls. This is especially difficult for glass objects, as for these, depth data are mostly disturbed, and in RGB images, occluded objects are still visible. Thus, in this paper, we propose to redefine the ground-truth for training RGB-based pose estimators in two ways: (a) we apply a transparency-aware multisegmentation, in which an image pixel can belong to more than one object, and (b) we use transparency-aware bounding boxes, which always enclose whole objects, even if parts of an object are formally occluded by another object. The latter approach ensures that the size and scale of an object remain more consistent across different images. We train our pose estimator, which was originally designed for opaque objects, with three different ground-truth types on the ClearPose dataset. Just by changing the training data to our transparency-aware segmentation, with no additional glass-specific feature changes in the estimator, the ADD-S AUC value increases by 4.3%. Such a multisegmentation can be created for every dataset that provides a 3D model of the object and its ground-truth pose.

## 1. Introduction

Household robotics is a challenging topic as it includes a lot of different tasks in a dynamic environment. For example, robots should be able to set the table, fetch objects, and place them in different places. To be able to manipulate any object, the robot needs to know that the object exists and where it is located in the world. This can be achieved, for example, with cameras perceiving color and depth information of the environment. Many works have been published about 6D pose estimation of opaque objects in different scenarios [1,2,3,4,5,6,7,8,9]. However, humans tend to use drinking devices, salad bowls, or decanters made out of glass; thus, the robot needs to be able to cope with transparent objects as well as opaque objects. There are two big differences between those objects: depth data are often unreliable, and clutter has a different effect. The problem with the depth data is that normal depth cameras rely on infrared light and laser range finders use high-frequency light. This may work fine with opaque objects where the light can be reflected but not really with transparent objects, as light just passes through and is either lost or reflected by objects or walls somewhere behind the actual object. This can distort the depth data quite a lot. There are different common approaches to tackle this problem: trying to reconstruct the depth data, as performed for example in [10,11,12], simply trying to solve the 6D pose estimation with stereo RGB images [13] or by just using single RGB images [14,15]. The latter is what our work focuses on.

**Figure 1:**
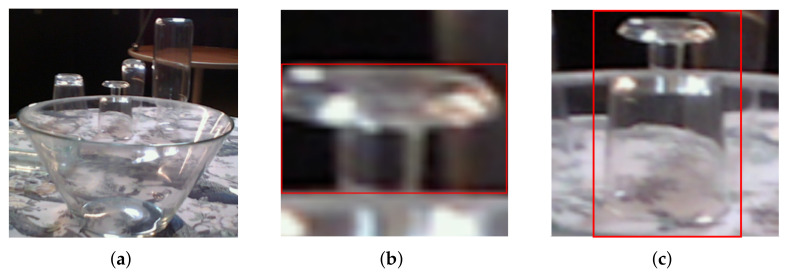
Example crops from a scene in the ClearPose [16] dataset with typical glass clutter, a wide overview (**a**) of the scene. The original bounding box (**b**) and an object transparency-aware bounding box (**c**), as well as their resulting regions of interest for the second stage.

Another difference between opaque and transparent objects is that clutter might not have the same visual effect. If an opaque object stands in front of another object, it covers the other object to a certain degree, and the object behind is only partly visible. With transparent objects, however, this is not always the case. A big glass object in front of another usually only covers small parts on its edges where the light reflects differently. But because its main part is just transparent, it is possible to see the object behind it quite well. Figure 1 highlights that the big salad bowl on the table (Figure 1a) stands in front of four other objects, but those are still visible, as a closer look on the wine glass in Figure 1c shows. Nevertheless, the ground-truth segmentation and the resulting bounding box Figure 1b are, by definition, only on the directly visible part of the object. This adds another difficulty for two-stage approaches. Usually, they first detect the object within a bounding box and scale the bounding box to a fixed size to then run a second network on that image. Now, if an object is largely occluded, the remaining part is scaled (Figure 1b), and consequently, the object has a much different scale than if not occluded. It might be quite helpful to prevent the wrong scaling and extend the bounding box to the whole object (Figure 1c) and teach the network that the hidden but visible part also belongs to the object. Thus, we propose to rethink the bounding box and segmentation for transparent clutter. We call this transparency-aware segmentation (TAS) and transparency-aware bounding box (TAB). The contributions of this paper are to

- modify ground-truth segmentation and bounding boxes to cover whole objects in cluttered transparent scenes,
- compare training with transparency-aware and original segmentation and its influence on the pose,
- apply our approach to a cluttered challenging glass scene using only RGB data without modifications specific to the glass dataset.

## 2. Related Work

As knowing the 6D pose of an object is probably essential for any robotic manipulation, there exist various methods to estimate it. They can be differentiated between direct methods that directly output a 6D pose such as PoseNet [17] and GDR-Net (geometry-guided direct regression) [18], or indirect methods that produce intermediate outputs and use RANSAC or PnP solvers to estimate the pose [1,15]. Another conceptual difference is the number of input images. Zebrapose [1] is a single-image approach that involves splitting the object into multiple regions with several sub-regions, interpreting every region as a certain class and as a multi-class classification problem before solving the resulting correspondences via a PnP solver. A multiple-view approach is, e.g., CosyPose [6], which uses a single image for initial object candidates, matches those candidates across other views, and globally refines object and camera poses. Another approach improving most results is to include depth data. FFB6D (full-flow bidirectional fusion network for 6D pose estimation) continuously fuses information from RGB and depth data within their encoder and decoder structured networks, followed by a 3D key-point detection and instance segmentation and least-square fitting [3].

All those methods, as well as most other work in this area, focus on texture-less, cluttered, and opaque objects; however, only a few works have been published specifically on transparent objects. As pointed out before, transparent objects face some further difficulties. There are some approaches using depth data, with some work on depth completion, as in [10], which uses affordance maps to reconstruct the depth data. Another approach is made by Xu et al. [11]. They try to reconstruct the depth image with by surface normal recovery from the RGB image and use this to calculate an extended point cloud. Using the RGB image and the constructed point cloud, another network estimates the final pose. Because of this depth problem there are also direct methods to use only RGB images like [14]. They adapt the idea of GDR-Net to use geometric features of objects and fuse those with edge features and surface fragments specified for transparent objects. From these fused features, a dense coordinate and confidence map is obtained and processed by GDR’s patch-PnP. An indirect approach is [15] by Byambaa et al. They also take only a single RGB image, but apply a two-stage approach where a neural network extracts 2D key points, followed by a PnP algorithm afterwards. These works have in common that they adapt their networks somehow to the specific characteristics of transparent materials, making them more specific and less suitable for general-purpose object pose estimation. We want to keep our network and training structure identical to previous work and rather change the ground-truth definition of the training data itself in a glass-dependent way. The idea is to enhance pose estimation on transparent objects without reducing the generality of the estimator.

In previous work, our group has proposed an indirect general approach for RGB and RGB-D images to estimate 6D poses [4] with a specific focus on symmetrical objects [19]. It splits the process into two stages, based on the idea that neural networks have been proven to predict well which object is where in the image and that mathematical algorithms can solve 2D-3D correspondence problems. In the first stage, a neural network is trained to predict a segmentation, an object image, and an uncertainty map of the predicted object image. This object image represents in each pixel the corresponding 3D point of the object in object coordinates in a second stage. These learned correspondences of the 2D pixels with the 3D object points within the object image are then solved by a generalized PnP algorithm, taking the uncertainty into account. This approach works well on symmetrical and opaque objects. In contrast to Xu et al., we do not change the structural training network and extend it with specific features for glass but rather reuse our general approach and simply adjust the training data to achieve better results on transparent objects.

Definitions for bounding boxes and segmentation of objects, which are necessary for most training processes in the area of 6D pose estimation, are hardly mentioned and discussed. Sometimes, it is implicitly displayed in figures [14] or encoded in the choice of the object detector [1] but rarely explicitly declared or discussed. In the case of bounding boxes, most works estimate axis-aligned bounding boxes, as in [20,21,22], and only few estimate object-oriented bounding boxes [23,24], but to our knowledge, there is no discussion about segmentation and the fact that one pixel can belong to more than one object, e.g., in the case of transparent objects or scenes seen through a window.

## 3. Approach

**Figure 2:**
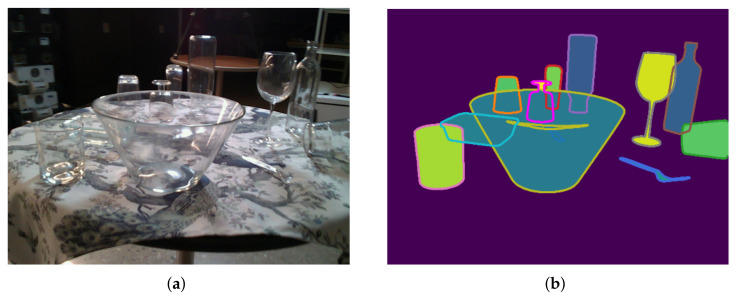
Example RGB image of the dataset (**a**) and the corresponding multisegmentation mask (**b**), where the fully colored pixels represent the original ground-truth segmentation, and the object border indicates that the objects continue. The pixels within the borders belong to all overlapping objects, not just to the object in front.

We want to investigate whether changing the training data considering the specialities of transparent objects enhances the pose estimation without having to adapt the network with special glass features. To achieve this, we use a multisegmentation. Conceptually speaking, we extend the normal segmentation mask, where each pixel is assigned to exactly one object or background, by assigning multiple objects to pixels in cases where clutter normally hides object pixels. The idea is visualized in Figure 2b. The implementation is performed as one segmentation image for each object individually within the image. Modifying and testing different training data with our indirectly working pose estimator requires different processes for training and prediction.

### 3.1. Training

**Figure 3:**
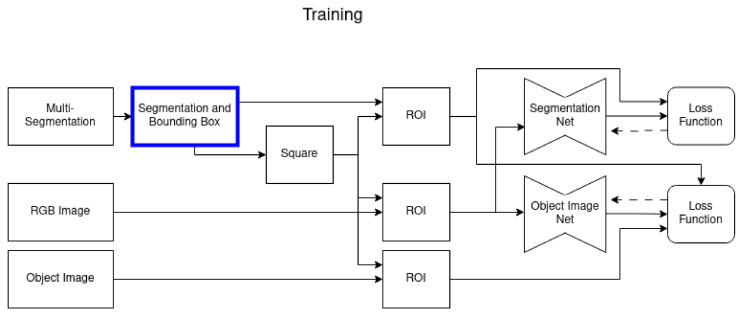
In the training phase, a multisegmentation is used to create our novel segmentation masks and bounding boxes, which is our main contribution (blue box). The dimensions of the bounding box are used to derive a squared region of interest. This region is used to crop the RGB input image as well as the ground-truth object image and segmentation. The two networks are trained independently, the segmentation network is trained to predict a segmentation map based on the defined ground truth, and the object image network is trained on the ground-truth object image. As its loss is on a pixel level, the ground-truth segmentation mask is used to only account for the loss for the pixels that are actually part of the object.

**Figure 4:**
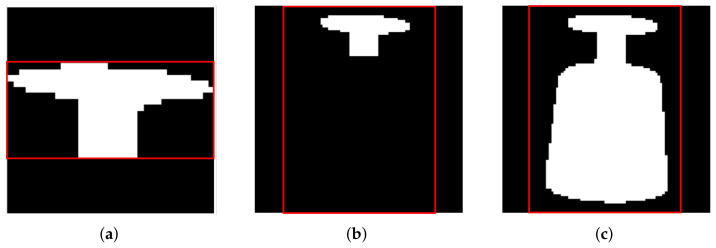
Different mask and bounding box sizes for the RGB image crops of Figure 1. (**a**) Displays the original mask and resulting bounding box, and (**b**) also uses the original mask but the transparency-aware bounding box. The right image (**c**) displays the transparency-aware segmentation and its resulting bounding box.

In the training processes, sketched in Figure 3, we consider three different variants in the segmentation and bounding box process. We want to investigate and assess the effect of a consistent size and scale of the object (transparency-aware bounding box), as well as of the transparency-aware segmentation definition. Figure 4 visualizes the three differences. In the first variant (Figure 4a), the original ground-truth mask and the resulting bounding box are used. The second variant (Figure 4b) also uses the ground-truth mask, but the transparency-aware bounding box (TAB) encloses the whole object plus an additional five pixels to every side. This ensures a consistent size and scale of the object and adds some more context around the object. The third training set is built by using the transparency-aware segmentation (TAS) mask and bounding box (Figure 4c), including even the formally occluded parts of the object. As glass is transparent, i.e., even though there might be some glass in front of the object of interest, the object of interest is still partly visible. Only the edges of the occluding objects really disturb the image. Having a fixed quadratic input size of the networks requires using the bounding boxes and calculating a region of interest that fits the networks’ needs. As in our previous work [4], we use the longest side of the bounding box to fit a square around the object (uniform scaling). The resulting ROI is used to crop the segmentation, RGB image, and our ground-truth object image. The images are either upscaled or downscaled to meet the second stage input size. Then, the two networks are trained independently of each other.

### 3.2. Prediction

**Figure 5:**
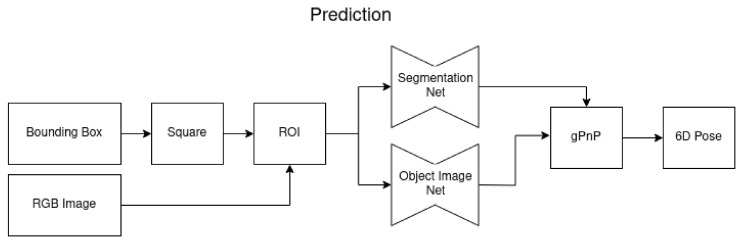
In the prediction phase, a bounding box for the object of interest is needed. It can come from any object detector. The dimensions of the bounding box are used to create a square around the object and use this as a region of interest for further processing. The incoming RGB image is cropped and scaled by this ROI and fed into a pre-trained segmentation and object image network. The object image network estimates the 3D points in object coordinates and an uncertainty map in pixel space. These two are used pixel-wise to solve the 2D-3D correspondence with the gPnP, while only the pixels are taken into account that belong to the object estimated by the segmentation network. This resolves into a 6D pose for this object.

At prediction time, an external set of bounding boxes of specific objects of the complete image is needed. To obtain these bounding boxes, any object detection algorithm can be used. This bounding box detection is a slightly different problem and out of the scope of this paper. Many methods were developed to find and classify specific objects within an image and produce bounding boxes [20,21,22,25,26]. These boxes can then be used to find more attributes like the color, materials, a text description of the object, and the 6D pose of the object. We focus on 6D pose estimation. Thus, for the prediction phase in our approach, we use the ground-truth boxes and the transparency-aware boxes, respectively, for the network training. For a real-life application, any object detector could be connected in front of our workflow, which is visualized in Figure 5.

## 4. Dataset

We chose the ClearPose dataset [16] to test our approach because it contains several real image scenes, in total over 350,000 annotated images, with many transparent, symmetric objects within cluttered scenes and annotated ground-truth segmentation maps and ground-truth poses. Other known available real-world datasets are ClearGrasp [27] and TransCG [28], which use plastic glasses instead of realistic real glass and include almost no glass clutter, making them uninteresting for our comparison. The complete ClearPose dataset includes several sets of scenes of different types, for example, normal objects placed jumbled on a table but also objects with filled liquors or with an additional translucent cover. We focus on set number 4. Set 4 includes about 43,000 RGB-D images with segmentation masks and poses. It consists of 6 scenes, all of them including the same 12 different objects: drinking glasses, bottles, bowls, and a knife and a fork, either made of glass or plastic, placed on a table. The difference between the scenes is their colorful tablecloth and the arrangement of the objects. This arrangement is random but physically correct, i.e., glasses are standing upright or upside down or lying flat on the table but do not balance on edges or lean on each other. The lighting seems to be realistic indoor room light, produced by fluorescent tubes and spotlights. To obtain the different images, a camera was moved around the table at different heights. This results in different light reflections and blurriness in the images. Sometimes, the unstructured environment of the lab where the images were taken is visible, depending on the camera position.

The ground-truth data were generated by a digital twin scene of the real scene and an estimated camera movement using ORB-SLAM3. With this information, the ground-truth segmentation and the true-depth image were rendered. This makes total sense for opaque objects but not so much for transparent objects. As visible in Figure 1, some pixels show different objects at the same time, where the bowl and the wine glass are behind it. The ground-truth segmentation is a gray image, with only one channel, encoding for each pixel if it is background or belongs to a specific object, but it does not represent multiple objects at the same pixel. Thus, for our experiment, we extended the ground-truth segmentation mask by using the provided 6D pose of the object and the provided 3D model and rendered a transparency-aware segmentation for each object in each image. For training, we used the included ground-truth and our own multisegmentation. Figure 2 shows one example image of the test set and our rendered multisegmentation.

## 5. Experiments

To evaluate our idea, we built expert networks for each object in Set 4, meaning we have one network for each object. In theory, this enables us to be unlimited in the number of objects we can process. However, in practice, this is limited by the memory needed. As we use these networks to evaluate Set 4 of the ClearPose dataset, we have 12 networks, one for each object, which we evaluate sequentially.

### 5.1. Network Architecture

**Figure 6:**
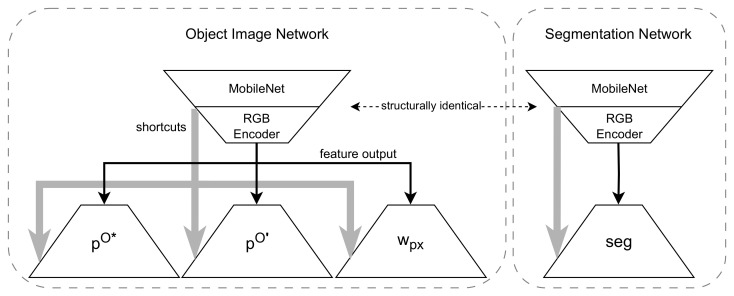
A more detailed sketch of our used network structure. The object image network has three heads. $p^{O*}$ and $p^{O^{\prime }}$ represent two different aspects of the object image points $p^{O}$, which is described in more detail in [19]. As this is just a technical detail, we will refer to $p^{O*}$ and $p^{O^{\prime }}$ together as an object image in the following. The pixel weight $w_{px}$ estimates the uncertainty in pixel space. In the first training stage, only the object images ($p^{O*}$ and $p^{O^{\prime }}$) are trained. In the second stage, the weights of the encoder are set and only the pixel weight head ($w_{px}$) is trained. The segmentation network has a simple common encoder–decoder with a shortcut structure. The layers of the encoder and decoder are listed in Table 1 and Table 2.

Our network is not new, but for the sake of completeness, we would like to present the used network architecture. In contrast to our approach in [19], we separate the original network into two networks for the segmentation and for the object image and train both of them independently. Although both have the same structural encoder (Table 1) with three layers of a pre-trained MobileNet and additional convolution layers, the decoder (Table 2) is different. This is schematically shown in Figure 6. The segmentation network predicts the segmentation, and the object image network predicts the object image, as well as an uncertainty map representing the uncertainty of each pixel in image space. The object image network from Figure 3 is in detail divided into one encoder and three heads as sketched in Figure 6. Table 2 shows the used structure of layers for each head, where they only differ in their output layer.

### 5.2. Training

We trained our networks on three NVIDIA A40 and two NVIDIA TITAN V using TensorFlow 2.11 and Python 3.8. To train the segmentation networks, we used a batch size of 64 and Adam as the optimizer with a fixed learning rate of 0.0001 and trained for 300 epochs. The object image network was trained with a batch size of 128 and used Adam as the optimizer with a fixed learning rate of 0.001 for 100 epochs on the object image and for another 200 epochs on the uncertainty map. The differences between the learning parameters resulted from the different computation powers. For each training set, we trained both networks for all objects, keeping all structural parameters the same, with no extra modification for any object or the training set. The networks have an input size of 224 × 224 and produce a segmentation mask, an object image, and an uncertainty map, each of size 112 × 112. Training and evaluation are performed on these three different training sets for each individual object to investigate the differences in 6D pose estimation. For a real-life application, an additional bounding box detector would be necessary.

As a metric, we use a symmetry-aware average point distance error (ADD-S) because almost all objects are somehow symmetrical. Only the knife and fork do not have a rotation symmetry axis. This is based on the pairwise average distance (ADD) [29] but includes the fact that points are ambiguous. Pure ADD would require the network to learn to guess the annotated rotation, which is arbitrarily set by the annotation. ADD-S is suitable to handle symmetrical and asymmetrical objects. We calculate the area under the curve (AUC) for thresholds from 0.0 m to 0.1 m, as in [16].

## 6. Results

We trained both networks separately. The segmentation network can not only give insights on their individual performance but also whether the network structure is suitable to identify the object at all. As stated before, the encoder structure of both networks is identical, and only the decoder differs.

### 6.1. Segmentation

The segmentation networks were trained for each object individually, each on the three different training sets: original, transparency-aware bounding box (TAB), and transparency-aware segmentation including the bounding box (TAS). Table 4 displays the recall and precision values on a pixel level averaged over all objects. The highest values were achieved by our transparency-aware segmentation. However, it is interesting that the transparency-aware bounding boxes have the lowest values. This might be because, for the additional context, without TAS, a lot more background pixels are added, producing a slight bias to background pixels as the precision is similar, but the recall values are worse. Overall, this also means that our network structure is, in general, suitable to detect object pixels within the image. Otherwise, the segmentation would not be as good.

### 6.2. 6D Pose Estimation

For the pose estimation, we use the prediction stage as displayed in Figure 5. The bounding boxes are either based on the original segmentation or the transparency-aware segmentation, depending on what data the respective network was trained on. The overall results on Set 4 are displayed in Table 5. Changing the ground-truth definition from original to TAS increases the AUC by 4.33% over all objects.

In order to compare our results with a reference value on this glass data set and to put them into a context, we compared them to Xu et al. [11] and FFB6D [3]. However, we do not claim to compete against their full approaches, as we use ground-truth bounding boxes without using any object detector. Even though [14,15] are methodologically more similar to our approach, we cannot compare ourselves to them as they use different data sets with little to no occlusion. Xu et al. developed a network with specific glass features, and FFB6D was a state-of-the-art network for pose estimation at the time when the dataset was published. Both have better results; however, both use RGB-D data, whereas our network only uses RGB data. In previous work, including depth data achieved an increase of up to 24% percent [4]. When FFB6D was trained and tested with ground-truth depth data, it achieved an improvement of almost 15%.

**Figure 7:**
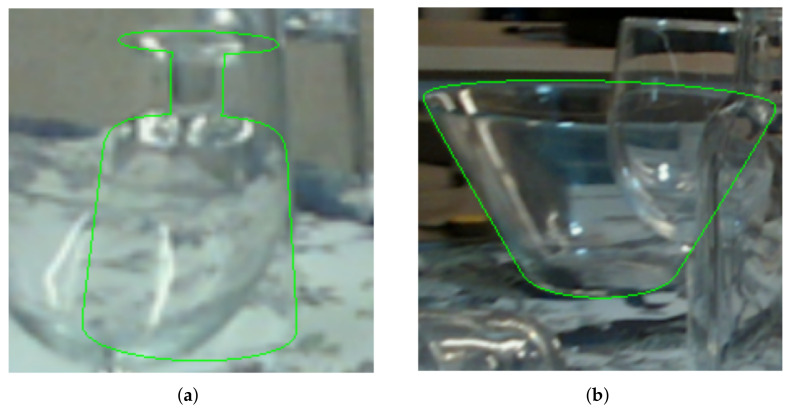
Two examples of difficult images where the estimated pose is rendered as a green contour on top of the RGB image. In (**a**), the wine glass is almost completely covered by another wine glass with reflections from the fluorescent tubes and has another glass in the back which also causes some edge distractions. The salad bowl (**b**) has multiple objects in the front and much background clutter.

To highlight the difficulty of the dataset, we show two example images and the resulting pose in Figure 7. The used networks were trained with TAS.

We evaluated our networks sequentially and a 6D estimation prediction step took 0.175 s on average. This includes the segmentation and object image network as well as the complete gPnP stage for all objects in the image. However, this excludes the time needed to load the networks, which would be necessary if the RAM is not big enough to keep all networks needed in memory. In our experiments, we needed around 42GB RAM to have all 12 networks running at the same time. It is worth noting that an additional object detector would also need some resources in a real-world application.

**Figure 8:**
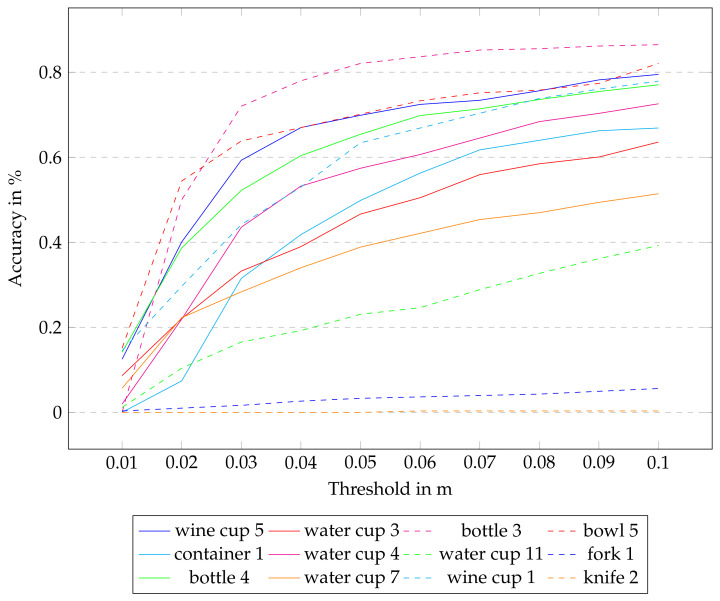
ADD-S accuracy-threshold curve of all 12 objects in Set 4 trained by using TAS.

To distinguish and have a more fine-grained result, in Figure 8 we calculated the ADD-S accuracy-threshold curve for all objects up to a threshold of 0.1 m, which is the same as the threshold for the ADD-S AUC. Interestingly, the results are quite different for different objects. It seems to work best on tall objects with a continuous symmetry axis. Not surprising is the fact that the knife and fork do not perform well. The special properties of these two objects are discussed in detail in Section 6.4. As our network could not deal with these two objects, we omit them in the next evaluation step.

### 6.3. Dependence on Level of Occlusion

**Figure 9:**
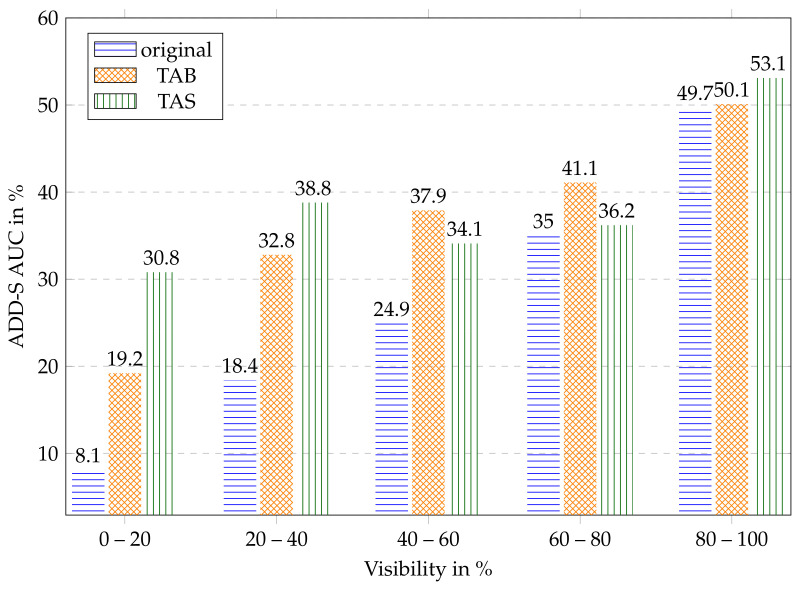
Results of the different training data distributed over the visibility of the objects, e.g., the bar of 0–20% visibility includes all images where the object is, based on the ground truth, only up to 20% visible.

**Figure 10:**
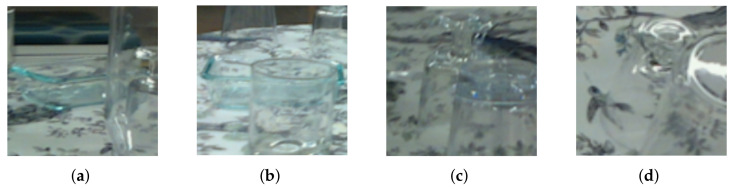
Example images of one bowl and wine glass with different visibility values around 50–70%: (**a**) bowl, 71% visibility; (**b**) bowl, 54% visibility; (**c**) wine glass, 55% visibility; (**d**) wine glass, 66% visibility.

To investigate the effect of the different training data, we performed an ablation study comparing the original bounding box and segmentation approach to our TAB and TAS. To obtain a more fine-grained result in relation to covered glass, we split the test data by their visibility using the percentage of the number of originally assigned ground-truth pixels to the number of TAS-rendered segmentation pixels (Figure 9). It is noticeable that our transparency-aware segmentation increases the AUC by 20% in the range of visibility up to 40% compared to the original. The strong effect leverages out as more of the object becomes visible, which makes sense because the training data is becoming similar. It is also noteworthy that the TAB also increases the AUC up to 14% in the range of 20–40% visibility and also performs better on all other images compared to the original. It seems like having the right object size and scale helps obtain a good object image, which encodes the geometric form, because even though the segmentation is worse than in the original approach, the 6D pose is significantly better, up to a visibility of 80%. This effect vanishes in the last scenario with 80–100% visibility. The difference in the aspect ratios of the two versions is not that big anymore as the original also encloses the whole object. The only real difference is the additional context. This was created by adding five pixels in each direction. The results indicate that this has only little influence as original and TAB are almost equivalent. An interesting effect occurs in the area 40–80%. The TAB achieves higher values than our TAS training data. Manual insights into test data in this range (see four examples in Figure 10) indicate that in these images, there is a lot of overlapping, disturbing the view of the object behind, as the edges reflect the light differently. This can result in actual hiding as in Figure 10a, where the right part is just not visible anymore, or in deforming the object as in Figure 10b, where the bottom of the bowl visually bends as seen through the glass in front of it. In these cases, it seems logical that the TAS might be too big, covering parts that do not belong to the object visually. It still achieves better results than the original training data.

### 6.4. Knife and Fork

**Figure 11:**
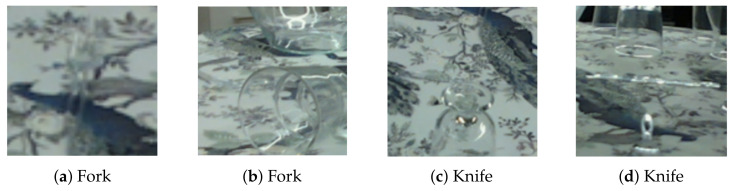
Example images of the fork (**a**,**b**) and the knife (**c**,**d**). Even if only the object is present, no distractions, (**a**), it can be really hard to see the fork (it is centered and points upwards). In (**b**,**c**) other glasses are within the bounding box and it is almost impossible to see the objects of interest. The right image highlights that even if the knife is visible, it has a completely different appearance because of reflections along the whole object.

There are two very challenging objects within the dataset: a transparent knife and fork (see examples in Figure 11). Both objects were hardly recognized by our network and did not profit from the different training settings. These two objects probably profit the most from the depth data, as they always lay flat on the table. Even if the depth camera does not recognize the object, it will detect the table underneath it. The fork has a maximum height of 15 mm and a knife of about 2 mm, meaning that the table depth is still a good approximation of the depth data for these objects. But without depth data, in RGB-only networks like ours, these objects could be anywhere in the room, making it really hard to predict. If we exclude these two objects, we obtain an AUC value of 43.26% with the original input and 48.36% for our transparency-aware approach.

## 7. Conclusions

We introduced a new way of defining segmentation masks and bounding boxes for transparent objects, being aware of the characteristics of glass and other transparent materials, to improve RGB-based pose estimation without having to adapt the network itself. With only a little effort, we render the multisegmentation mask, which can be used to create transparency-aware segmentation and bounding boxes. This can be achieved independently of the dataset if 3D object models and ground-truth poses are available. Using these instead of the original training data in the training process benefits the 6D pose estimation of a given RGB estimator if the objects can be recognized by the network at all. This is particularly true when there is strong occlusion and objects are only partially visible according to the original definition.

One downside of our approach is that a 3D object model and a ground-truth pose have to exist to generate the transparency-aware segmentation mask. This is not as easily applicable if the objects of interest are parameterizable or articulated without a fixed model. However, most work is still required on fixed and stable objects; thus, it seems like a good and easy option to increase the accuracy of 6D pose estimation for transparent objects just by adapting the training set. The success of TAB suggests the hypothesis that presenting the objects in the right scale, even if they are occluded in the training data, as it is with the original segmentation, might also help 6D pose estimation on opaque objects.

## Figures and Tables

**Table 1:** RGB encoder structure.

Layer Name	Nr. of Output Channels	Filter Size	Stride	Output Size	Nr. of Layers
MN block_3_project_BN	32	-	-	28 × 28	-
Conv1	100	5 × 5	2	14 × 14	1
Dense1	64	-	1	14 × 14	12 × 3
Conv2	128	1 × 1	1	14 × 14	1
Dense2	64	-	1	14 × 14	12 × 3
Conv3	128	1 × 1	1	14 × 14	1
Conv4	70	5 × 5	2	7 × 7	1
Dense3	70	-	1	7 × 7	6 × 3
Conv5	140	1 × 1	1	7 × 7	1

MN block_3_project_BN—the used output layer of the MobileNet v2 version of keras, ConvX—a 2D convolution operation, DenseX—a custom dense block as described in Table 3.

**Table 2:** Decoder structure.

Layer Name	Nr. of Output Channels	Filter Size	Stride	Output Size	Nr. of Layers
Up1	140	-	-	14 × 14	1
Concat2	268	Conv3 (Enc.) & Conv5 (Enc.)		14 × 14	1
Conv6	50	3 × 3	1	14 × 14	1
Dense4	25	-	1	14 × 14	12 × 3
Conv7	50	1 × 1	1	14 × 14	1
Up2	50	-	-	28 × 28	1
Concat3	82	Dense4 & MN block_3_project_BN		28 × 28	1
Conv8	32	3 × 3	1	28 × 28	1
Dense5	16	-	1	28 × 28	6 × 3
Conv9	32	1 × 1	1	28 × 28	1
Up3	32	-	-	56 × 56	1
Concat4	56	Dense5 & MN block_2_project_BN		56 × 56	1
Conv10	24	3 × 3	1	56 × 56	1
Dense6	12	-	1	56 × 56	4 × 3
Conv11	24	1 × 1	1	56 × 56	1
Up4	24	-	-	112 × 112	1
Concat5	56	Dense6 & MN bn_Conv1		112 × 112	1
Conv12	16	3 × 3	1	112 × 112	1
Dense7	8	-	1	112 × 112	4 × 3
Conv13	16	1 × 1	1	112 × 112	1
Conv ($p^{O*}$/$p^{O^{\prime }}$/$w_{px}$/seg)	3/3/4/1	1 × 1	1	112 × 112	1

Enc.—Layer from the Encoder, ConvX—a 2D convolution operation, ConcatX—a concatenation layer, UpX a 2D upscaling layer, DenseX—a custom dense block as described in Table 3, MN X—the used layer of the MobileNet v2 version of keras.

**Table 3:** Dense block structure.

Layer Name	Nr. of Output Channels	Filter Size	Stride	Output Size	Nr. of Layers
ConvI	input channels	1 × 1	1	input size	1
ConvII	input channels	3 × 3	1	input size	1
Concat	Input Layer & ConvII			input size	1

ConvX—a 2D convolution operation, ConcatX—a concatenation layer. The number of channels and the output size depends on the layer before, where the input channels and input size is determined.

**Table 4:** Results of the test set segmentation on a pixel level. Best results in bold.

	Original	TAB	TAS
recall	83.64%	79.13%	**89.53%**
precision	93.10%	93.82%	**97.22%**

**Table 5:** Results of the test set. The results from Xu et al. and FFB6D are taken from [16].

	Xu et al. [11]	FFB6D [3]	Original	TAB	TAS
ADD-S AUC	45.23%	43.45%	36.27%	38.27%	40.60%
without knife + fork	-	-	43.26%	45.85%	48.36%

## Data Availability

Publicly available datasets were analyzed in this study. This data can be found here: https://github.com/opipari/ClearPose accessed on: 24 December 2023.

## Abbreviations

The following abbreviations are used in this manuscript:

ADD-S	Symmetry-aware average point distance
AUC	Area under the curve
TAB	Transparency-aware bounding box
TAS	Transparency-aware segmentation
ROI	Region of interest
